# Designing a trapping strategy to aid Giant African Snail (*Lissachatina fulica*) eradication programs

**DOI:** 10.1371/journal.pone.0203572

**Published:** 2018-09-07

**Authors:** Amy Roda, Mary Yong Cong, Bryce Donner, Katrina Dickens, Amy Howe, Shweta Sharma, Trevor Smith

**Affiliations:** 1 Miami Laboratory, Science and Technology, Plant Protection and Quarantine, Animal Plant Health Inspection Service, United States Department of Agriculture, Miami, Florida, United States of America; 2 Division of Plant Industry, Florida Department of Agriculture and Consumer Services, Gainesville, Florida, United States of America; 3 University of Florida, Department of Landscape Architecture, Gainesville, Florida, United States of America; University of Minnesota, UNITED STATES

## Abstract

In pest eradication programs, traps can directly reduce pest populations; however, their application to gastropod programs remains relatively unexplored. The South Florida Giant African Snail, *Liassachatina fulica* (Pulmonata: Achatinidae), eradication program allowed a realistic evaluation of their utility. Field studies were conducted to determine the best bait, barrier and trap for use during the eradication program. Immature and adult snails were attracted to banana fruit and a commercially produced bait but only the commercially produced bait did not attract non-target and pest mammals. Four commercially produced traps and 4 barriers were field evaluated for snail retention efficacy. Snails escaped all traps and trap/barrier combinations but the rate of escape ranged from 10–100% after 24 hrs. Laboratory studies confirmed that snails can survive crossing a 5 cm barrier of copper tape, salt, insect stickem or antifouling paint. In the laboratory study snails did not cross copper sulfate but they crossed the barrier in the field. Adding salt to traps as a means to retain snails reduced the number of snails trapped. Laboratory studies confirmed that dry salt decreased the number of snails entering traps and snails did not enter traps when the salt was dissolved in water. Two trap types and the commercial bait were selected for a large-scale program test. For three months, trapping along with hand collection and pesticide application were conducted on 114 properties in five locations. Traps caught snails when surveys and regular pesticide applications on the same properties did not detect them. On 21 occasions snails were only found in traps, and both immature and adult snails were caught. This study showed that traps could be effectively deployed in an eradication program and they could capture snails that may have escaped other control measures.

## Introduction

The Giant African Snail, (*Lissachatina fulica* (Bowdich Gastropoda: Pulmonata: Achatinidae) is considered one of the world’s worst invasive alien species [1]. The snail attacks agricultural and native plants, vectors plant pathogens, and threatens human health while functioning as a host in the life cycle of rat-lung worm, *Angiostrongylus cantonensis* [2]. Native to continental Africa, Giant African Snails (GAS) have spread throughout the tropical and subtropical worlds [3–5]. Large-scale and costly eradication programs are often undertaken, which include surveys, hand collection, pesticide applications and debris removal [6–9]. A snail traps could aid eradication programs as a tool to detect populations at low levels [10, 11]. In addition, traps could continuously remove snails particularly from areas difficult to access and where the presence of pets and farm animals precludes pesticide applications.

Integrating a trapping system into a snail eradication program would require overcoming challenges imposed by both the pest’s biology and the need to conduct program activities on private properties. The trap should allow entry and retain snails that range in size from 5–200 mm [2]. The bait used should preferably attract both immature and adult snails, which have been shown to change their feeding preferences during development [2]. In an urban program, the trap and bait must be child, pet and environmentally-safe and have minimal negative impact on homeowner landscapes. Furthermore, the trapping system needs to fit the eradication program by being readily available for large-scale deployment, easy to service, and cost effective.

Snail traps are available commercially or can be made from locally available materials [12–14]. These systems use a food bait alone or combined with a killing agent such as salt or a pesticide like metaldehyde [12, 15–17]. Baits containing yeast or fermented products have been found to attract more GAS compared to fresh plant material or the pesticide metaldehyde alone [16, 17]. Barriers such as salt [13], copper sulfate [16], insect stickem [18] and copper tape [18–20] have been shown to prevent GAS and other gastropod species from entering crops but their use in combination with a trap remains relatively unexplored. Surface trapping using a non-baited refuge mat trap is a simple method recommended by researchers, consultants and molluscicide manufacturers to monitor slugs [21]. Studies have shown that a large number of slugs are trapped using a refuge despite the high availability of alternative shelters provided by surrounding vegetation [22]. In terms of operating an eradication program, a simple refuge trap would be an appealing option for ease of deployment as well as for having a minimal impact on non- target animals.

The GAS eradication program in Florida, USA provided an opportunity to field test the use of snail traps on a large scale. We evaluated different trapping systems on their ability to capture snails, ease of deploying and servicing them in the field, and their acceptable use in residential yards. The highest rated baits and traps were then tested for three months during the Florida GAS eradication program. Final evaluation and recommendations are made for including trapping as part of a snail eradication or monitoring program.

## Materials and methods

### Field sites

Field studies were conducted on residential properties located in Miami-Dade County, Florida from October 2011–December 2012. The properties were located in the most heavily infested quarantine zones [8]. These were established by the United States Department of Agriculture (USDA) and Florida Department of Agriculture and Consumer Services (FDACS) GAS joint eradication program [23]. Property owners within the quarantine area signed release forms granting full permission for accessing and conducting control efforts. No endangered or protected gastropod species were present on the quarantine properties. The properties were all subject to an eradication protocol, which entailed visual surveys, hand collection, debris removal, and pesticide treatments at least once every two weeks [8, 9]. From October 2011 through October 2012 pesticide applications were limited to 1% iron phosphate granular bait (Sluggo^™^ Loveland Products, Inc. Greeley, CO). Beginning October 2012 5% boric acid (Niban^®^ Nisus Corp. Rockford, TX) was also used but limited to monthly applications [9]. Study sites were located on properties with the highest snail populations based on previous program surveys and on visual detection of actively foraging snails the day the experiment was started. In all experiments baits, trap types and trap and barrier combinations were placed linearly 1.5 m apart beneath mixed ornamental plantings on the boarder of residential properties.

### Laboratory studies

Studies were conducted at the FDACS Biological Control Laboratory containment facility, Gainesville, Florida. The GAS colony, established with approximately 1,500 hand-collected snails from South Florida, was fed organic romaine lettuce (*Lactuca sativa* L.; Asterales: Asteraceae) and provided with a cuttlebone for calcium [9]. Experimental snails were divided into 3 classes based on their developmental stages: neonates (7–20 mm), juveniles (21–47 mm) and adults (>47 mm) [8, 24]. Twenty four hours prior to experimentation, snails within the median size range of each class were starved by holding them in vented plastic containers (18x28x10 cm). All experiments began at sunset (approximately 18:00) and were evaluated 12 hr later. Environmental conditions were maintained at 80 ± 5 °C; 75 ± 10% RH.

### Baits

In the first field experiment, banana fruit and banana pseudo-stems were evaluated as potential baits for the trapping system. Cavendish (AAA cultivar group) bananas (*Musa acuminata* Colla) were purchased from local grocery stores. A 150–200 g piece held in an open petri dish (60 mm diameter) was placed in the center of a 25x25x12 cm plastic box that had a 1–1.5 cm layer of table salt (Morton Iodized Salt, Chicago, IL) on the bottom. The box was covered with a lid to limit the amount of rain entering the trap, but had two 5x15 cm square openings cut into each side of the container to allow snail access. *Musa* (AA) ‘Lady Finger’ banana pseudo-stems were obtained from a local Miami, FL organic garden. One meter lengths of pseudo-stems 10–15 cm in diameter were cut into 25–30 cm longitudinal sections. These sections were cut in half and a 3–5 cm diameter portion of the center was removed to serve as refuge for the snails. The two halves were held together with plastic flagging tape. Four salt box traps with banana fruit and 4 pseudo-stems were placed on 2 residential properties (25.67157, -80.42677 and 25.52201, -80.40996). After 24 hours, all snails found in the trap or pseudo-stem were removed and the number and sizes recorded. For 3 days, the traps and pseudo-stems were inspected with the locations re-randomized after the data were collected to account for potential positional effects on the number of snails trapped (n = 24).

In a second field experiment, banana fruit and a commercially produced bait (Snail Buster, Environmental Services, Tallahassee, FL) were tested. The Snail Buster bait was mixed 3:1 tap water to bait, and 100 ml of saturated bait was placed in a petri dish held in the center of the salt box trap. In this experiment the petri dish with the banana fruit was placed in a 10x10x15 cm hardware wire mesh basket then placed in the center of the salt box trap to limit access of non-target mammals. The study was conducted on 2 residential properties (25.67157, -80.42677 and 25.67097 and -80.42639) with 2 salt box traps baited with banana fruit and 2 traps baited with Snail Buster bait on each property. For 3 days the traps were inspected and their location re-randomized. The experiment was repeated with fresh banana and Snail Buster bait the following week (n = 24).

### Traps

**Fig 1:**
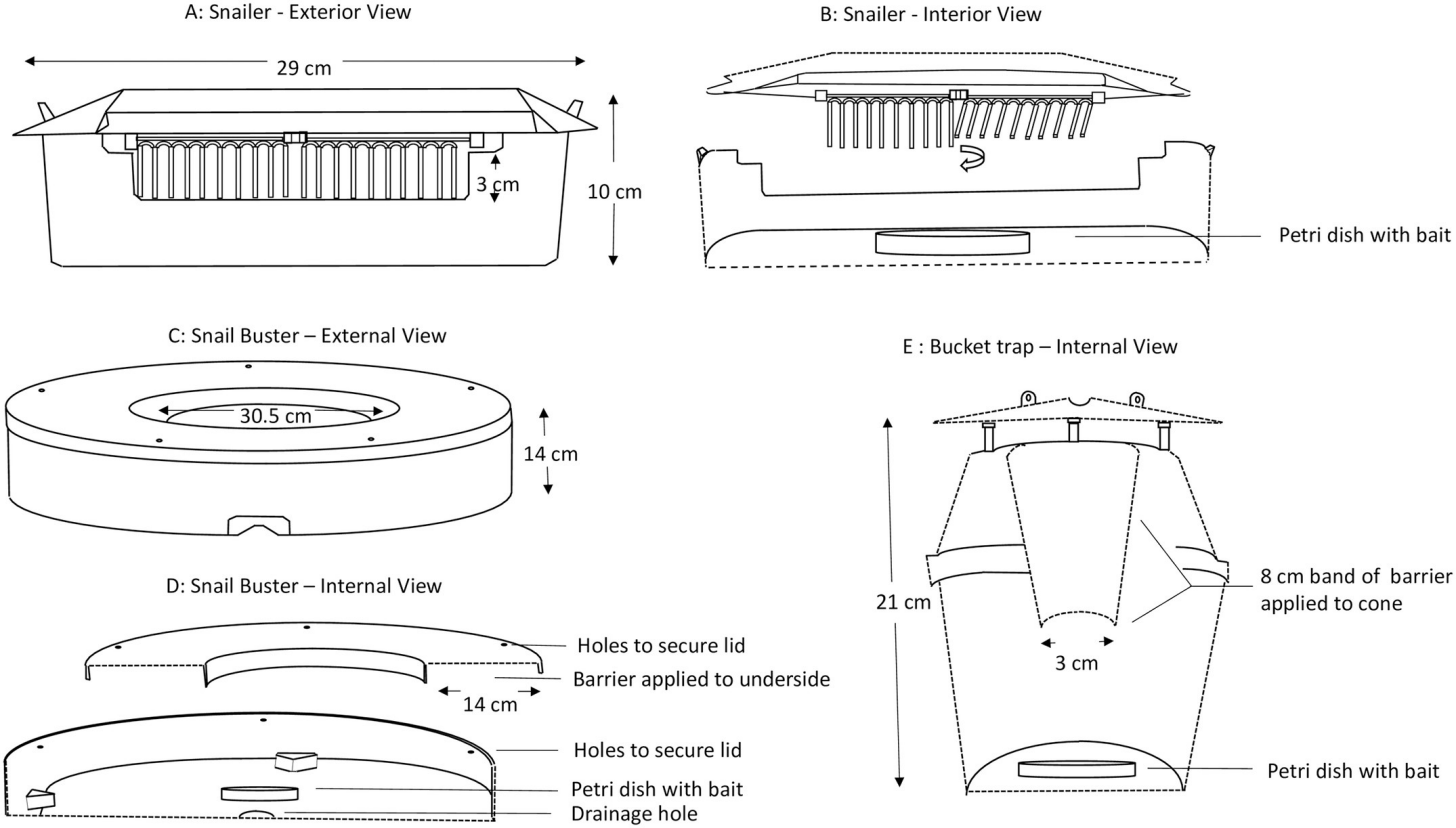
Three commercially available snail traps tested in the South Florida (USA) Giant African Snail eradication program: Snailer (A-exterior view, B-internal view), Snail Buster (C-exterior view, D-internal view) and bucket trap (E-internal view).

Four commercially available traps, Snailer Snail and Slug Trap^™^ (American Organic Products, Ventura, CA, Fig 1A and 1B), Snail Buster ^™^ (Environmental Services, Tallahassee, FL, Fig 1C and 1D) and yellow top and white bottom universal moth bucket traps (Great Lakes IPM, Inc., Vestaburg, MI, Fig 1E) and slug mats (Liphatech, Milwaukee, WI, not pictured) were baited with 100 ml of Snail Buster bait placed in a petri dish at the bottom of the trap. Slug mats were made from a 49x49 cm piece of silver insulated reflective fabric designed to create a high moisture area under the mat. On 2 properties (25.819305, -80.198731 and 25.819348, -80.198799) 2 of each of the 4 trap types were placed 1.5 m apart linearly under a mixed ornamental planting that ran along the border of each property. Traps were randomly assigned a location in the late afternoon (15:00–16:00) and evaluated every 24 hr for five days. All trapped snails were marked with a unique number using a white oil-based paint marker (Sharpie, Oak Brook, IL) and returned to the trap. The trap location was re-randomized daily along the hedge to avoid any potential location bias and allow the snails equal access to the traps (n = 20).

### Barriers

Four types of barriers were tested in the field: copper tape (Corry’s Slug and Snail copper tape barrier, Central Garden & Pet Company, Atlanta, GA), table salt, insect sticky trap spray (Stickem Special^®^, J.T. Eaton & CO., Inc., Twinsburg, Ohio) and copper sulfate pentahydrate crystals (QC LLC, Cape Girardeau, MO). Barrier placement in the traps varied with only one type of barrier tested per trap. As above all traps were baited with 100 ml of Snail Buster bait, the traps with or without a barrier were randomly assigned a location along the border of the property and inspected every 24 hr. All snails trapped were counted, measured, marked and returned to the trap, and the trap locations were re-randomized each day. For studies with the Snail Buster trap, salt or copper sulfate was glued to the underside of the lid, or copper tape was attached to the inner lip (Fig 1D). Two Snail Buster traps with one of the three barriers were tested on one property (25.819469, -80.198702) for 3 days (n = 6). Three paired experiments were conducted with bucket traps combined with barriers on separate weeks. The first compared insect stickem spread on the lower 8 cm of the inner cone to a bucket trap with a clean cone. The second paired experiment tested the effect of salt glued to the lower 8 cm of the inner cone compared to a clean cone. The third experiment compared traps with table salt (100 ml) placed in the bottom of bucket to traps without salt. For each of the three experiments, four traps with and without the barriers were placed on one of three properties (25.819492, -80.198011, 25.819469, -80.198702 and 25.819474, -80.199104 respectively) and evaluated every 24 hr for five days (n = 20).

Laboratory studies with the barriers were conducted to validate field observations that GAS crossed them. The four barriers tested in the field (copper tape, table salt, copper sulfate, insect stickem) along with two additional barriers, copper oxide (Alpha Chemicals) and antifouling paint (Rust-Oleum Marine, Vernon Hills, IL 25% Cuprous Oxide A.I.), were evaluated. Arenas were established by placing a 5 cm border of the barrier around a 30x40 cm white paper board (n = 7). Two snails of each size class were placed in the center of the arena. Twelve hours later the numbers of snails that crossed the barrier and if they were dead or alive were recorded.

### Effects of salt in traps on snail behavior

Laboratory experiments were conducted to evaluate the effect of table salt (100 ml), salt (100 ml) dissolved in water (100 ml) and no barrier on the number of snails entering traps. In the first study, 2 snails of each size class were placed in the center of a 60x60x60 cm mesh arena (BugDorm ^™^, MegaView Science, Taiwan). A Snailer trap with salt only or salt dissolved in water was placed on opposite sides of the tent. Each trap was baited with the 100 ml of Snail Buster bait held in a petri dish in the center of the trap (n = 5). After 12 hours the number of each size class of snail found in the trap was recorded. In a second laboratory experiment only the juvenile size class was tested. As above, Snail Buster bait was placed in the center of a Snailer trap. The juvenile snail was given one of two paired choices: dry table salt vs. no salt or water saturated salt vs. dry salt. After 12 hours, the number of snails in each trap was counted (n = 18).

### Eradication program field test

From October–December 2012, a large-scale evaluation of the optimal trap, bait and barrier combination was conducted by program staff as part of regular eradication operations. One hundred and fourteen properties with the highest population of GAS based on previous surveys were selected for the trial. In October both bucket and Snailer traps were deployed, and in November-December only Snailer traps were used. Snail Buster bait prepared as above was placed in the center of the trap. No barriers were used. The bait was replaced each week or when more than 50% was consumed. Each day all snails were removed and transported to the laboratory where they were counted and measured.

### Data analysis

Continuous data were tested for normality [25]. Non-parametric data were analyzed with a Kruskal–Wallis test and consequently with a post-hoc procedure of multiple comparisons of all treatments [26]. The choice study data were analyzed with a G-test to test the null hypothesis that the observed distribution of visits with an equal number of visits to each of the treatments was random [27].

## Results

### Baits

Giant African Snails were attracted to banana fruit, banana pseudo-stems and the Snail Buster bait (Table 1). 90% more snails were caught with the banana fruit in a salt box compared to the banana pseudo-stem. In the December 2011 study, 85% more snails were collected in traps with Snail Buster bait compared to banana fruit baited traps. Both immature and adult snails were attracted to the banana fruit and Snail Buster bait. Only immature snails were found in the banana pseudo-stems.

**Table 1:** Decision matrix for optimal bait to test in the South Florida (USA) Giant African Snail eradication program.

Bait	Oct 2011 Mean ± SE (#snails/trap/day)	Dec 2011 Mean ± SE (#snails/trap/day)	Attracted snail size (mm)	Bait advantages^+^	Bait disadvantages^-^
Banana pseudo-stem	0.38±0.17 (0–4)	-	8–30	A	F
Banana fruit	2.0±0.68 (0–21)	0.33±0.33 (0–2)	5–50	B, D, E	G, H
Snail Buster bait	-	2.5±1.28 (0–18)	8–75	B, C, D, E	G, I

^+^Advantages: A-Provides a refuge with high humidity; B- Commercially available; C-Consistent and known quality; D-Miminal preparation required, E-All size classes trapped

^-^Disadvantages: F-Limited availability; G-Rotted within 2–3 days; H-Non-targets removed bait, I-Dried

The banana fruit rapidly degraded in the field and became infested with maggots within 3 days. Additionally, the banana fruit was frequently missing, and tracks in the salt indicated opossum and rodents as likely consumers. When the banana fruit was placed in a wire basket to prevent non-target access, the snails utilized the wire basket to climb down from the lid of the salt trap box to the bait, and thereby escaped touching the salt. Like the banana fruit, the Snail Buster bait became infested with maggots within 3–5 days. However, non-target mammals did not remove the bait. The difficulty of maintaining banana fruit in the traps and the likely possibility of attracting vermin to residential yards precluded wide-scale program use. Although the banana pseudo-stems attracted the snails, there was no means to retain the snails. In addition, obtaining a large and consistent supply of banana pseudo-stems was difficult, which would make program-wide use of them impractical.

### Traps

**Fig 2:**
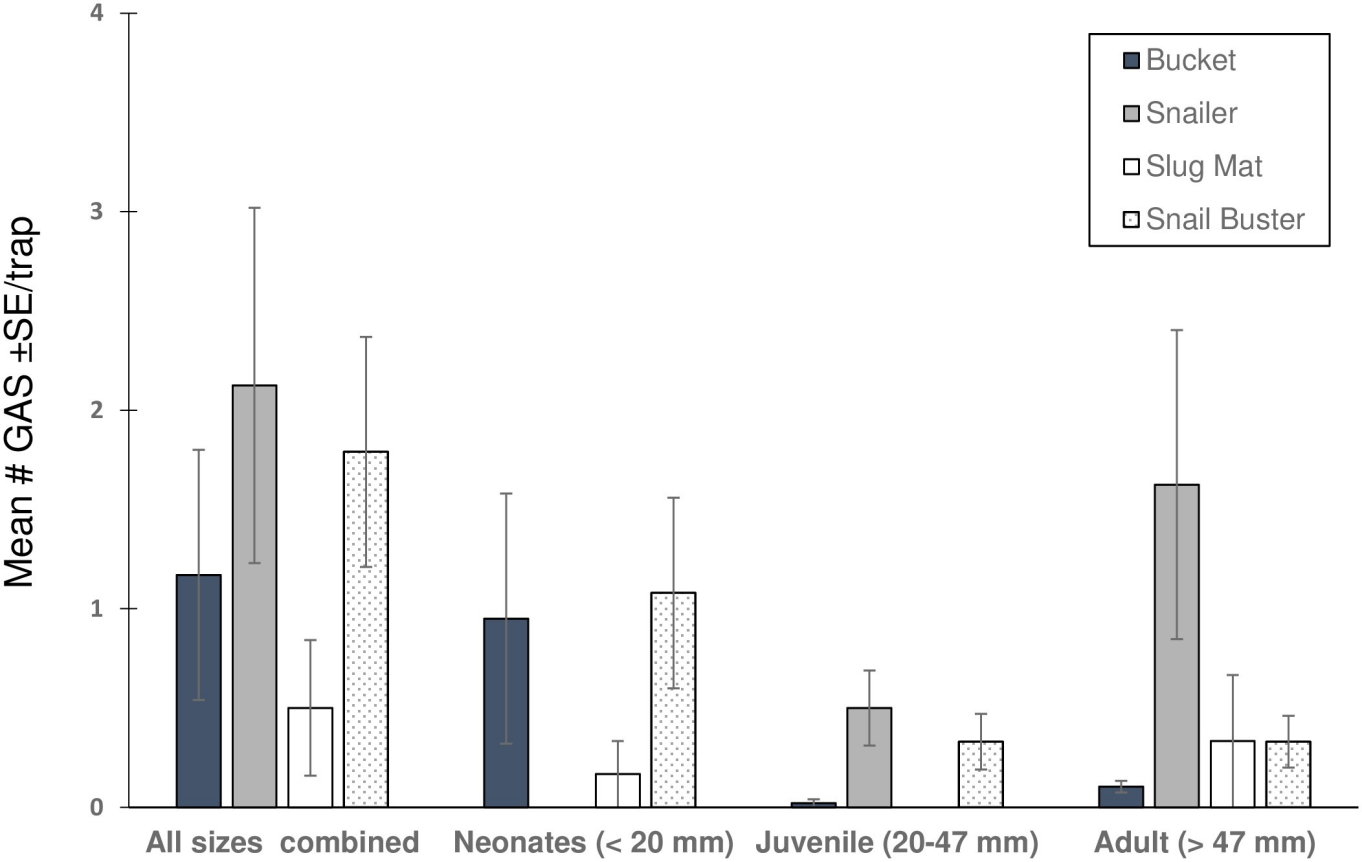
The total mean (±SE) and size class mean (±SE) number of Giant African Snails field trapped using 4 different commercially available traps (n = 20).

Snails were collected from all trap types (Fig 2). There were no differences in the total number of snails trapped (Χ^2^ = 4.24, df = 3, p = 0.24). However, there were differences in the number trapped based on size class. More adult snails (>47 mm) were collected from the Snailer trap compared to the bucket trap (z = 2.72, p = 0.007). The number of adult snails trapped with the Snailer and Snail Buster traps were not different. No neonate snails (<20 mm) were found in the Snailer and no juvenile snails (21–47 mm) trapped with the slug mat.

Snails were able to escape all traps (Table 2). All snails from the slug mat were missing after 24 hr as this trap had no system to retain the snails. The Snail Buster and bucket traps had the lowest number of snails escaping with only 10 and 11% of the marked snails leaving the traps. However, snails missing from an individual trap ranged from 0 to 100%. Smaller snails were observed escaping by directly climbing through the main entrance and any holes used to fasten the trap together or for drainage like those needed in the Snail Buster trap. The larger snails escaped traps by reaching from the bottom of a trap to the entrance side wall and pulling themselves through the opening. With the Snailer and Snail Buster traps, large snails were observed to reach the bait and feed without pulling their shell into the trap. An adult snail originally captured in a Snailer trap escaped and was recovered from two different Snailer traps on two subsequent days. Apparently, the snail had learned to lift the swing door mechanism to leave the trap after feeding on the bait.

**Table 2:** Decision matrix for optimal trap to test in the South Florida (USA) Giant African Snail eradication program.

Trap	Trap size (height x width cm)	Trap opening (cm)	Trapped snail size range (mm)	Water accumulation	Marked snails escaping in 24 hr	Escape route*	Design advantages^+^	Design disadvantages^-^
Snailer	10 x 29	3 x 18	31–75	moderate	40%	1,2,3	A,C,E	F
Snail Buster	14 x 58	30.5 diameter	8–65	high	10%	1,2,3	D,E	G, H
Slug mat	49 x 49	NA	25–40	low	100%	1,4	B	H
Bucket trap	21 x 18	3 diameter	10–66	low	11%	1	A,B,C,D	I

* Escape route: 1-Crawl back through entrance, 2-Reaching into trap and not completely entering the shell, 3-Crawling through holes (drainage and those to secure lids), 4-No barriers or retainers so all snails escaped

^+^ Design advantages: A-Small size, inconspicuous, B-Completely covered reducing debris and water entry, C-Limited accessibility by non-target mammals, D- Complex lid inhibits movement out of trap, E-Large number of snails captured

^-^ Design disadvantages: F- Free movement of small snail sizes, G-Easily flooded and created a mosquito environment or caused salt to wash out, H-Very large so difficult to place in concealed locations where snail populations persist, I-Entrance may limit largest snails.

### Barriers

**Fig 3:**
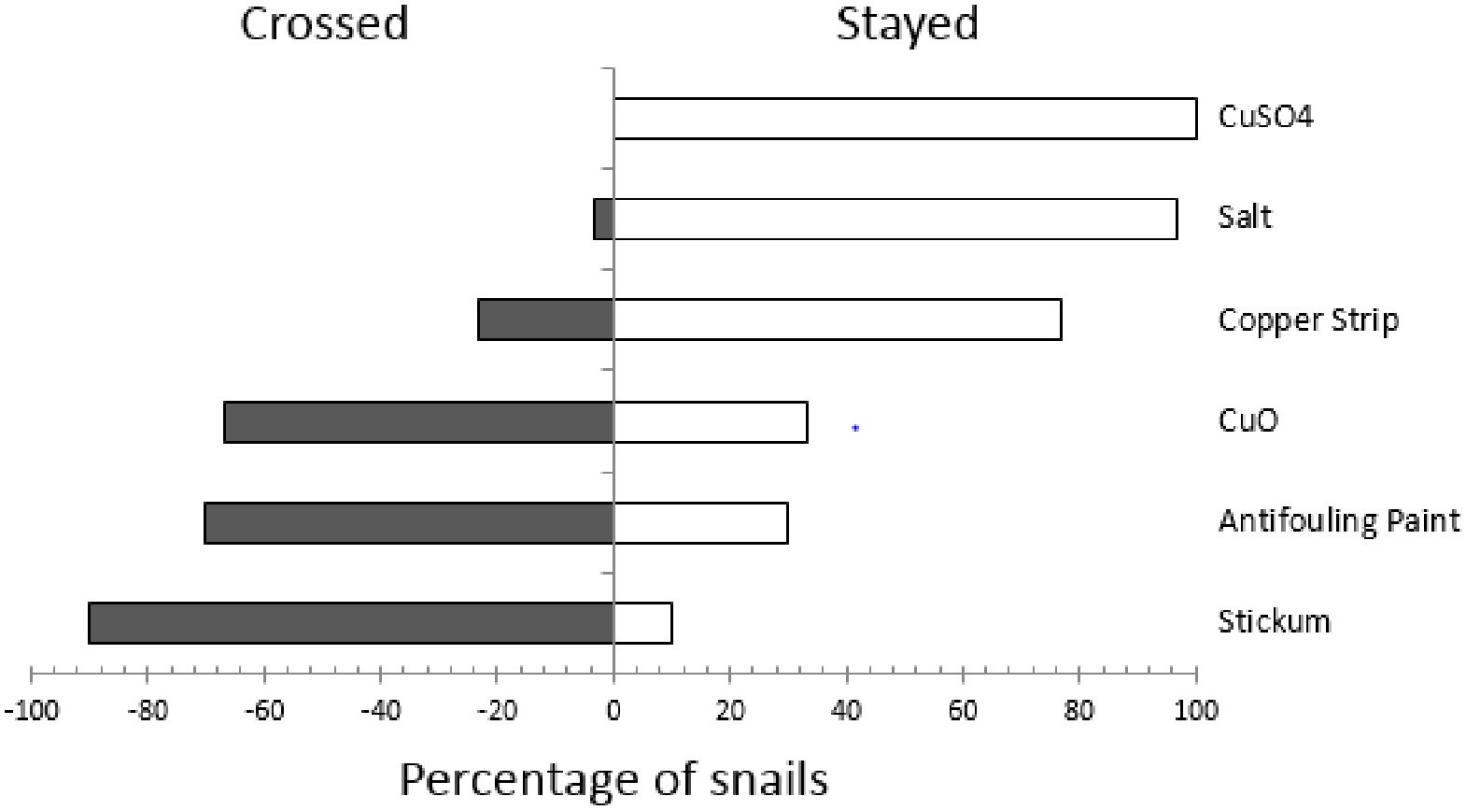
The percentage of Giant African Snails crossing a 5 cm barrier of copper sulfate (CuSO_4_), table salt (salt), snail and slug copper tape (copper tape), copper oxide (CuO), marine antifouling paint (antifouling paint) and insect stickem trap (stickem) after 12 hr under laboratory conditions (n = 7).

In laboratory studies, the type of barrier affected whether or not the snail crossed (Χ^2^ = 89.82, df = 5, p = <0.001, Fig 3). No snails crossed the copper sulfate barrier and 8 snails were found dead touching the barrier. With salt, 1 juvenile snail successfully crossed and survived. Twenty percent, which included all size classes, crossed and survived the copper strip. More snails of all size classes crossed the insect spray adhesive, copper oxide, and antifouling paint than remained and 4, 11 and 5% respectively died after crossing.

**Fig 4:**
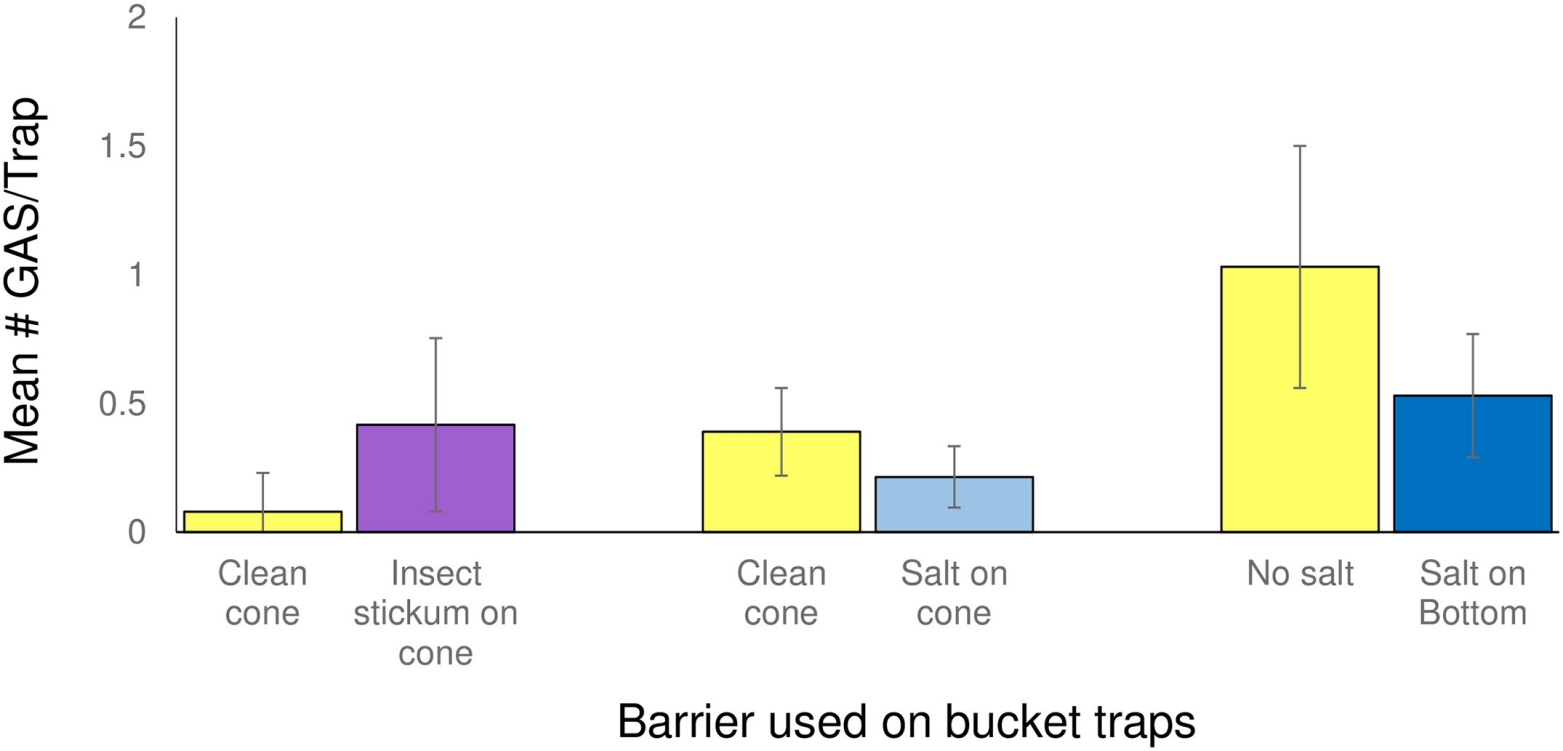
The mean (±SE) number of Giant African Snails (GAS) field captured in bucket traps (n = 20) without a barrier (clean cone and no salt) and with barriers (insect stickem, salt on cone or salt on the bottom).

In the field, snails escaped Snail Buster traps when copper tape or a coating of copper sulfate or salt was placed on the trap lid. Marked juveniles and neonates in addition to adults were found outside the trap. With the copper sulfate and salt coatings, the high humidity and rain affected the adhesive, causing quantities of the barrier (approximately 25–50%) to dislodge. Slime trails with traces of the blue copper sulfate were seen on the top of the Snail Buster lids, indicating the snail was able to avoid the lethal effects of the barrier to an extent to allow escape. However, the majority of the slime trails ended with a dead snail. In field studies, adding barriers to bucket traps, whether stickem or salt, did not increase the number of snails trapped (Fig 4). No marked snails escaped the bucket traps that had salt glued to the cone or placed on the bottom, whereas 25% of the marked snails left the traps with stickem. Interestingly, the bucket trap without a barrier (the control treatment) retained 100%, 66% and 100% of the marked snails in the stickem, salt on the funnel and salt on the bottom experiments, respectively.

### Effects of salt in traps on snail behavior

In field studies, when the salt in the box, Snail Buster, and bucket traps became saturated with rain water, fewer snails were found (Roda, unpublished data). In addition, 50% more snails were trapped in bucket traps without salt (Fig 4: clean cone, no salt) than when salt was added to the bucket trap (Fig 4: salt on cone and salt on the bottom). In the field study where Snailer and bucket traps with salt on the bottom were compared to traps with no salt, snails were found only in the no salt traps. However, only 3 snails were trapped in two traps (2 juveniles in a Snailer trap and 1 juvenile in a bucket trap) during the study. In addition, rainwater entered the traps. These observations prompted laboratory studies where the presence and amount of water and salt could be controlled.

**Fig 5:**
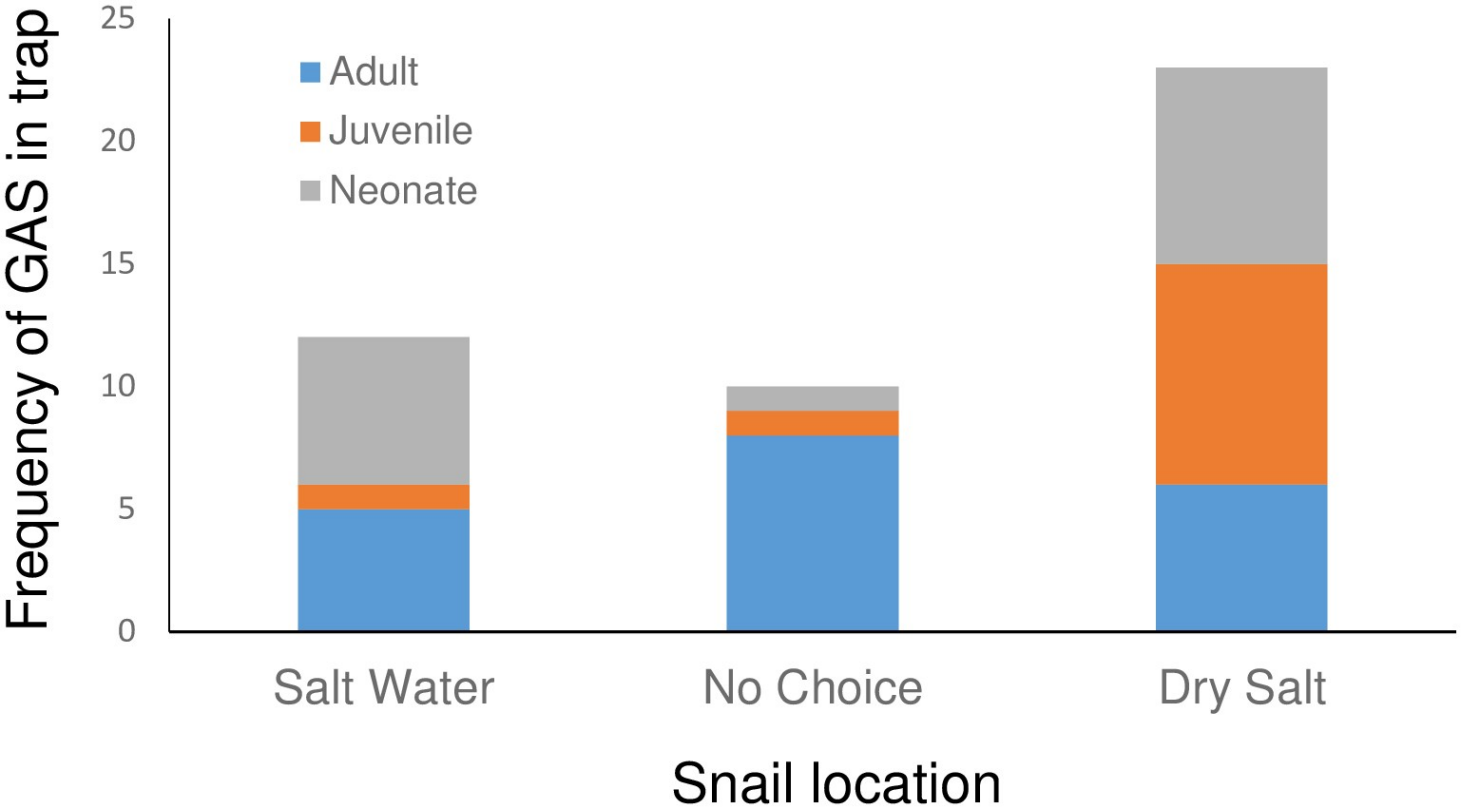
The frequency that 3 size classes of Giant African Snails (GAS), adults (> 47 mm), juvenile (21–47 mm) and neonate (<21 mm), entered baited Snailer traps with either water-saturated table salt or dry table salt.

**Fig 6:**
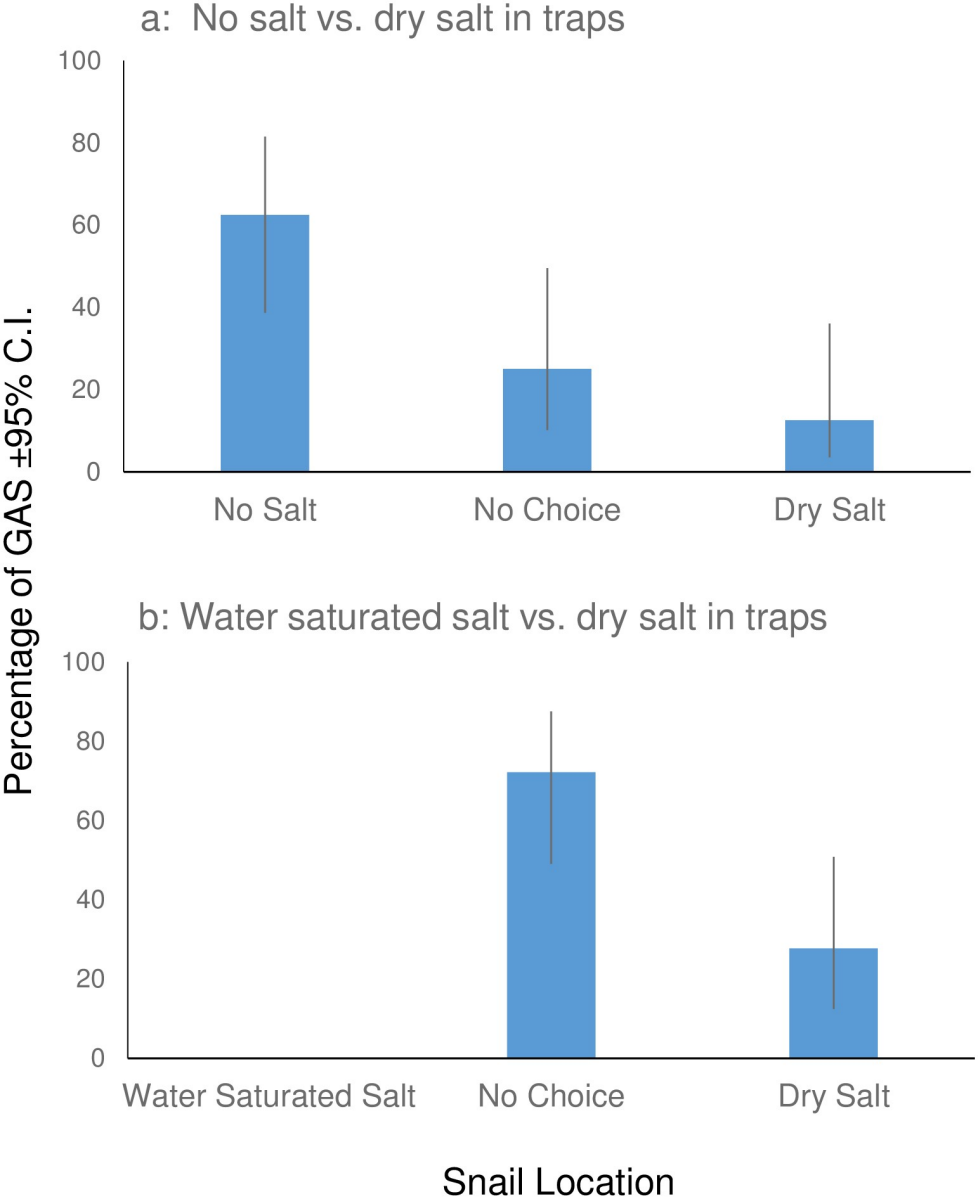
The percentage (± 95% C.I.) of juvenile (21–47 mm) Giant African Snails (GAS) that entered baited Snailer traps with (a) no salt or dry salt (n = 18) and with (b) water-saturated salt or dry salt (n = 18).

Laboratory results showed salt decreased the number of snails entering traps (Figs 5 and 6). When 3 size classes were evaluated (Fig 5), juvenile snails were the only size class to enter the dry salt traps more frequently than the water saturated salt traps, or not entering a trap altogether (Χ^2^ = 11.64, df = 2, p = 0.003). Adults and neonates showed no differences. Adults were observed reaching and feeding on the bait without touching the salt or salt solution or entering the trap. In the second experiment where only juvenile snails were tested, snails entered traps with no salt more frequently than when salt was on the bottom of the trap (Χ^2^ = 6.5, df = 2, p = 0.038; Fig 6A). The inhibitory effect of the salt was increased when water was added to the salt (Fig 6B). No snails entered the traps with the saltwater and more snails did not make a choice (Χ^2^ = 12.82, df = 2, p = 0.0016) than entered the traps with salt or the saltwater solution.

### Eradication program field test

The Snail Buster bait was selected for testing in the eradication program because the bait had several advantages. The bait attracted all GAS size classes and trapped them consistently more than the other baits (Table 1). The bait was commercially available, easy to deploy and produced with consistent quality. In addition, non-target mammals did not remove the bait, which was important factor to keep the trap attractive to snails as well as not to attract vermin to homeowner yards.

The trap system selected capitalized on the standard operating practices of the eradication program. On a weekly basis, the eradication crews surveyed and treated the properties, returning to the area the following days until all infested properties were treated [8]. Hence, servicing the traps on a daily or bi-weekly process would not entail additional trips to the area. This allowed the simplest system: a trap with no barrier and a bait. Despite the continuous pesticide treatment and hand removal of snails, populations remained in areas where yard debris, sheds, and fencing made access difficult. Thus, the traps selected were ones that could easily fit into confined spaces such as under buildings or between fences. The smaller traps also had the benefit of being less conspicuous on homeowner properties.

Two traps were recommended for testing in the eradication program: the bucket and the Snailer trap. Despite being one of the smallest traps (Fig 1, Table 2), the Snailer traps caught and retained a large number of snails (Fig 2). This trap was particularly efficient at collecting adults and juvenile-size snails but not neonates that could pass through the forks on the flap door. Therefore, the bucket trap was also included in the program trial. The bucket traps caught the largest number of neonate snails (<20 mm) but also trapped larger snails (Fig 2). In addition, the design of the bucket trap enhanced snail retention as no snails were observed reaching from the outside of the bucket trap and feeding on the bait, as was seen with other designs.

In the October trial, the Snailer traps caught significantly more snails than the bucket traps (Table 3). Both traps retained all size classes for 24 hr despite the lack of barriers. However, signs of feeding on the bait as well as frass present inside indicated that snails had escaped. Snails were often found <1 m from the traps along with these apparent signs of escape (Roda and Cong, unpublished data). Although in these cases the traps did not retain the snails, they drew them out of refuges to areas where they could be found and collected.

**Table 3:** The number of adult (>47 mm), juvenile (21–47 mm) and neonate (<20 mm) Giant African Snails caught in bucket and Snailer traps placed on 114 infested properties located in South Florida (USA). The traps were serviced daily for one month.

Trap	Adults		Juveniles		Neonates	
	Mean (SE)	Total	Mean (SE)	Total	Mean (SE)	Total
Bucket	0.02 (0.01)	15	0.06 (0.01)	38	0.01 (0.004)	4
Snailer	0.05 (0.01)	38	0.09 (0.01)	66	0.04 (0.01)	26

In the second test, where only Snailer traps were deployed, one adult, one juvenile and three neonates were trapped in November and two adults, six juveniles and no neonates were trapped in December. This lower number corresponded to a dramatic decrease in snail populations on the property [8]. Most importantly, traps caught snails on 21 dates, and on 21 different properties during the same period when pesticide applications followed by hand collection did not uncover snails.

## Discussion

In pest eradication programs, traps can directly reduce pest populations; however, their use in gastropod programs remains relatively unexplored [10, 11, 28]. The large scale eradication of GAS in South Florida allowed a realistic evaluation of utilizing traps to aid the program. This study showed that traps could be deployed on a large scale and they could capture snails even when pesticide application and hand collection on the same properties did not uncover snails until subsequent days. The traps also revealed areas where snail populations were escaping control measures. After finding a snail in a trap, staff intensified pesticide application, debris removal and visual surveys in the surrounding area. Thus, the snail traps also functioned to detect residual populations so that they could be targeted for control, which is an essential component to successful eradication programs [11].

Snails more readily entered traps with large access openings and that were around their foraging height. The size of the bucket trap entrance and its location (32 mm, Fig 1c) may have prohibited or deterred larger snails from entering the trap. The ease of access also frequently facilitated escape. In the field, large snails were observed stretching to climb out of the trap entrance or to reach the bait without fully entering the trap. Most interestingly, marked snails found in a neighboring Snailer trap on subsequent readings showed the snails could open the swing door and freely move to another trap. This study showed that a trapping system for GAS still requires further research to balance the need to provide the snail easy entry into the trap with mechanisms to retain them until the trap is serviced.

The use of barriers did not stop snails from escaping the traps. In the field, GAS readily crossed or circumvented all barriers tested in this study. Studies have shown that the width of a barrier needs to be in proportion to the body size of the terrestrial gastropod to prevent their access to plants [29]. GAS crossed a 3 cm salt barrier but they could not cross a 6 cm barrier [16]. Similarly, Prasad et al. (2004) found that a 5 cm band of salt prevented GAS from reaching protected plants. Although, the original size of the barriers used in our field studies was large (≥8 cm), the adhesives did not withstand the very hot, humid conditions, and frequent rains that dislodged the barrier from the trap during this study.

Müller and Weber (1999) found in field tests that terrestrial gastropods detected the weakest points of the barriers and repeatedly used the path to reach the protected plants. In this study, slime trails with traces of the blue color of copper sulfate provided evidence that the snails found a path out of the trap. Fortunately, both laboratory studies and field observations indicated that the snails most likely died from contact with these barriers. Other field studies have shown salt and copper sulfate to be effective barriers [16] and that rain reduced their performance [30–32].

Traps used in areas with public contact should be designed so the bait and any killing agents do not injure or come into contact with other animals or humans [30, 33]. Although copper sulfate was found to be effective in this and other studies [16], we did not recommend wide-scale testing to the eradication program. The chemical is labeled dangerous as the product can cause skin irritation, serious eye damage, and is corrosive upon ingestion [34]. This tool may be appropriate in commercial settings where children and pets would not have access to the product.

Salt traps have been recommended to capture GAS [12, 15, 18]. This study showed that GAS will enter salt traps. However, our laboratory studies showed that the salt acted as a repellant and the repellency increased when the salt was saturated with water. Therefore salt traps were not recommended for the eradication program. In addition, the field trials showed that snails entered traps containing only bait and that a large proportion of the marked snails remained in a trap for multiple days. In this situation, the trap could be serving as a protected refuge as well as a feeding location [35]. By not using salt, the negative effects of the snail avoiding the trap as well as the time and cost to add the barrier were curtailed.

Ongoing eradication program can provide excellent research opportunities [11]. In this study we evaluated available snail trapping systems and the interaction with the pest’s behavior to uncover problems that hinder the use of traps in an eradication program. We found a bait that attracted both adult and immature snails and a trapping system that could be deployed on a large scale. However, the recommended trapping system was not adopted by the eradication program after the 3 month test despite the traps being effective in trapping snails when other control measures conducted on the same property did not reveal snails. The cost in time to service the traps, the frequent indication of escape, the high number of empty traps, and the fast, unpleasant decay of the bait were all given as reasons for not continuing. Clearly, further research is needed to optimize both the attractant and the trap. We tested traps and baits designed for capturing a wide range of gastropod species. In eradication programs, the most effective trapping systems are often developed specifically for the target species [10]. Synthetic chemical lures made from pheromones, if discovered, or mimicking food attractants, are needed in combination with a trap where GAS can easily enter but not escape. Although there is a need for further development, this study showed that traps could be a useful tool in a snail eradication program.

## Supporting information

S1 File Bait, trap and barrier field and laboratory data. (XLSX)

S2 File Giant African Snail program evaluation of snail trapping systems data. (XLSX)
